# L1CAM deployed perivascular tumor niche promotes vessel wall invasion of tumor thrombus and metastasis of renal cell carcinoma

**DOI:** 10.1038/s41420-023-01410-4

**Published:** 2023-04-04

**Authors:** Zonglong Wu, Yaqian Wu, Zhuo Liu, Yimeng Song, Liyuan Ge, Tan Du, Yunchong Liu, Li Liu, Cheng Liu, Lulin Ma

**Affiliations:** 1 Department of Urology, Peking University Third Hospital, Beijing, 100191 P.R. China; 2 School of Nursing, Beijing University of Chinese Medicine, Beijing, 100191 P.R. China; 3 Department of Urology, Shanghai General Hospital, Shanghai Jiao Tong University School of Medicine, Shanghai, 200080 P.R. China

**Keywords:** Renal cell carcinoma, Focal adhesion, Chemotaxis

## Abstract

The survival of tumor cells in the bloodstream, and vasculature adhesion at metastatic sites are crucial for tumor metastasis. Perivascular invasion aids tumor cell self-renewal, survival, and formation of metastases by facilitating readily available oxygen, nutrients, and endothelial-derived paracrine factors. Renal cell carcinoma (RCC) is among the most prevalent tumors of the urinary system, and the formation of venous tumor thrombus (VTT) is a characteristic feature of RCC. We observed high expression of L1CAM in the VTT with vessel wall invasion. L1CAM promotes the adhesion, migration, and invasion ability of RCC and enhances metastasis by interacting with ITGA5, which elicits activation of signaling downstream of integrin α5β1. L1CAM promotes ADAM17 transcription to facilitate transmembrane ectodomain cleavage and release of soluble L1CAM. In response to soluble L1CAM, vascular endothelial cells release several cytokines and chemokines. Endothelial-derived CXCL5 and its receptor CXCR2 promote the migration and intravasation of RCC toward endothelial cells suggesting that crosstalk between endothelial cells and tumor cells has a direct guiding role in driving the metastatic spread of RCC. LICAM plays a crucial role in the invasive ability of RCC, and regulation of L1CAM expression may contribute therapeutically to preventing RCC progression.

## Introduction

Renal cell carcinoma (RCC) is among the most prevalent tumors of the urinary system, and venous tumor thrombus (VTT) formation in the renal vein or inferior vena cava is common in 4–10% of patients with locally advanced RCC [1]. VTT can be in the form of a free-floating tumor thrombus or may adhere to the vessel wall and continue to invade, suggesting vast cellular heterogeneity of RCC [2]. Patients with vessel wall invasion have significantly worse postoperative survival outcomes and suffer from higher tumor burden, tumor stage, grade, and lymph node involvement [3].

The adhesion of cancer cells to vascular endothelial cells is crucial for tumor metastases. Interestingly, tumor cells persist in the perivascular niche at the site of metastasis during the early stages of metastasis [4, 5]. Perivascular localization facilitates ready access to oxygen, nutrients, and endothelial-derived paracrine factors that promote tumor cell self-renewal, proliferation, and survival, ultimately leading to metastases [6–8]. The process of metastatic growth within the perivascular niche by hijacking the existing vasculature for further growth is called vascular co-option. Tumor cells that communicate closely with vascular endothelial cells can adhere to the endothelial cell surface and grow around blood vessels. Cancer cells that fail to adhere to the vasculature often struggle to colonize, survive, and metastasize [9, 10]. However, the molecular basis of vessel-tumor interaction has not been completely elucidated, partly because of the limited number of experimental models. Adhesion of the VTT to the vascular wall provides a model for studying the crosstalk between RCC and the vascular endothelium.

L1CAM is an immunoglobulin superfamily member that contains six immunoglobulin-like domains (Ig domains), five fibronectin repeats (FN repeats), a transmembrane domain, and a highly conserved cytoplasmic domain [11]. Aberrant expression of L1CAM in different human cancers is often associated with poor survival prognosis. This correlation is closely related to the ability of L1CAM to enhance tumor cell proliferation and invasion and to maintain tumor stemness [12–14]. The LICAM upregulation was observed in the invasive front in non-small cell lung cancer and colon cancer cells and lung cancer tissues around blood vessels [15–17]. Several studies have highlighted the role of L1CAM in the transendothelial migration of cancer cells, which is considered a marker and driver of metastasis-initiating cells [18, 19]. Contrary, the downregulation of L1CAM was observed in pancreatic ductal carcinoma [20]. The disintegrin and metalloprotease (ADAM) family cleaves the transmembrane ectodomain of L1CAM extracellularly, releasing a soluble L1CAM ectodomain (L1CAM-ECD) of ~200 kDa from the cell surface, which has been detected in serum samples from patients with ovarian and uterine cancers [21, 22]. L1CAM-ECD is functionally active and binds to cells in an autocrine or paracrine manner and promotes cell migration, metastasis, and angiogenesis [23, 24]. However, the exact role and mechanism of LICAM in tumor metastasis and angiogenesis in RCC remain unclear.

In our study, we observed a high expression of L1CAM in RCC-VTT tissues with vessel wall invasion. L1CAM promotes RCC cell adhesion, migration, and invasion. In addition, soluble L1CAM mediates crosstalk between RCC and vascular endothelial cells and promotes the directional migration of RCC to vascular endothelial cells. Our study suggests that L1CAM is involved in the malignant process in RCC cells, and the regulation of its expression may contribute to the prevention of RCC progression and therapeutic efficacy.

## Results

### L1CAM was highly expressed in RCC-VTT tissues with vessel wall invasion

**Fig. 1:**
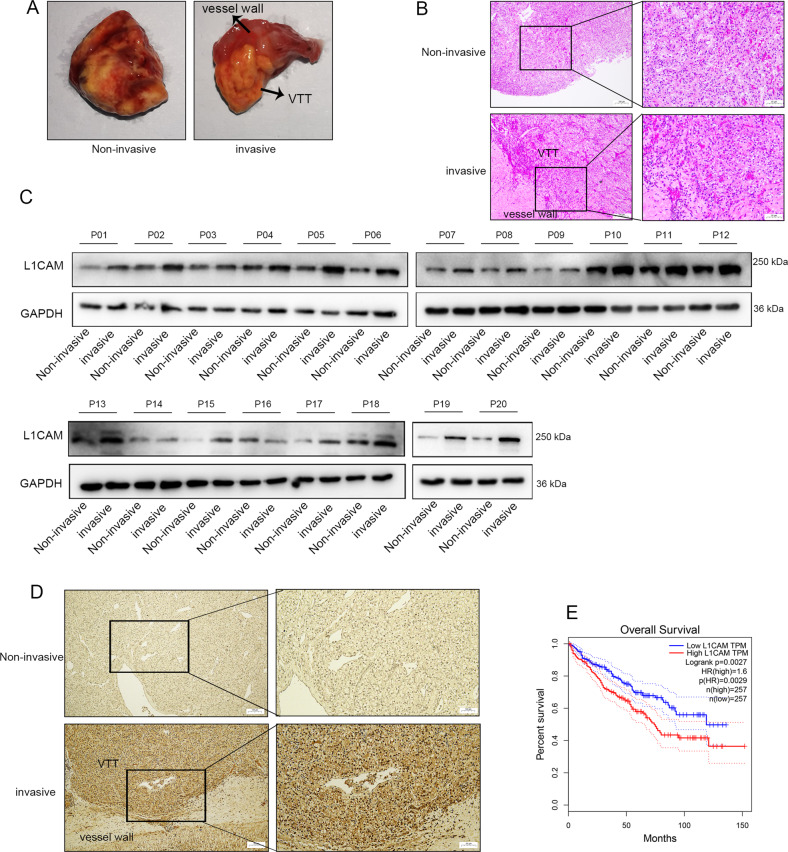
L1CAM was highly expressed in RCC-VTT tissues with vessel wall invasion. Twenty pairs of noninvasive and invasive VTT tissue adjacent to the vessel wall tissue samples were collected. **A** Specimens of noninvasive and invasive VTT tissue samples adjacent to the vessel wall. **B** H&E staining of VTT specimens. VTT venous tumor thrombus. **C** L1CAM protein expression in 20 pairs of VTT tissue samples. P patient. **D** Representative images of IHC staining of L1CAM in noninvasive and invasive VTT tissue adjacent to the vessel wall sample (40× and 200× magnifications). **E** Survival analysis of TCGA data using GEPIA online software.

Twenty pairs of samples for noninvasive VTT tissue adjacent to the vessel wall and VTT tissue invading the vessel wall were collected, and HE staining was performed to determine the VTT adherence and vein wall (Fig. 1A, B). Western blotting showed that VTT tissues with vessel wall invasion had high expression of L1CAM (Fig. 1C). Additionally, IHC analysis of the VTT tissue with vessel wall invasion showed strong positive staining for L1CAM. Furthermore, it was observed that positively stained RCC cells were predominantly located on invasive borders rather than inside tumor cell clusters (Fig. 1D). Analysis of TCGA data using GEPIA (http://gepia.cancer-pku.cn/) revealed that high expression of L1CAM was significantly associated with poor survival prognosis in patients with RCC patients (Fig. 1E).

### Suppression of L1CAM expression decreased the adhesive, migration, and invasion ability of RCC cells

**Fig. 2:**
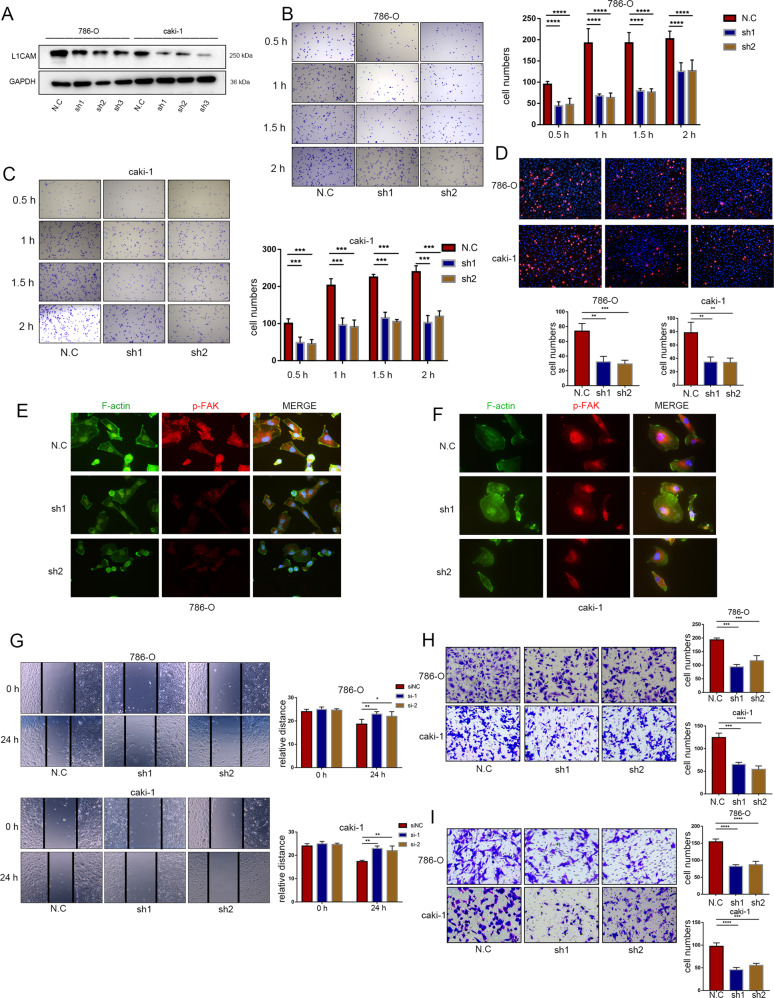
Suppression of L1CAM expression decreased the adhesive, migration, and invasion ability of RCC cells. **A** After infection with lentivirus, the effect of L1CAM knockdown was evaluated in 786-O and Caki-1 cells using western blotting. **B**, **C** An ECM adhesion assay was used to assess the effects of L1CAM knockdown on the cell-ECM adhesion ability of 786-O and Caki-1 cells. Adherent cells were quantified in five randomly chosen fields. **D** A tumor-endothelial adhesion assay was used to assess the effects of L1CAM knockdown on the cell-endothelial adhesion ability of 786-O and Caki-1 cells. The adherent cells were quantified in five randomly chosen fields. **E**, **F** Immunofluorescence of F-actin stress fiber formation and focal adhesion complex assembly in 786-O and Caki-1 cells after L1CAM knockdown (400× magnification). **G** Wound healing assays were performed on 786-O and Caki-1 cells to evaluate the effect of L1CAM knockdown on scratch repair activity. **H**, **I** 786-O and Caki-1 L1CAM knockdown cells were evaluated by Transwell migration and invasion analysis. The migrated and invaded cells were quantified in three randomly chosen fields. All data are presented as the mean ± SD. *P < 0.05, **P < 0.01, ***P < 0.001, and ****P < 0.0001.

Lentiviral shRNA was used to suppress the expression of L1CAM in RCC cell lines 786-O and Caki-1 (Fig. 2A). Through extracellular matrix (ECM) adhesion assay, we observed that L1CAM knockdown reduced the adhesion of RCC cells to ECM (Fig. 2B, C). Tumor-endothelial adhesion assay showed L1CAM knockdown reduced the adhesion of RCC cells to vascular endothelial cells (Fig. 2E). The formation of stable focal adhesion complexes is related to actin cytoskeleton structure; it mediates cell-to-cell and cell-to-ECM adhesions [25]. The p-FAK level (Tyr397) is critical for promoting the focal adhesion complex and improving actin cytoskeleton dynamics [25]. It was observed that L1CAM knockdown inhibited F-actin stress fiber formation and focal adhesion complex assembly (Fig. 2E, F), migration, and invasion of the RCC cells (Fig. 2G–I), as indicated by immunofluorescence, wound healing, Transwell migration, and invasion assays.

### Overexpression of L1CAM promotes the adhesion, migration, and invasion abilities of RCC cells

**Fig. 3:**
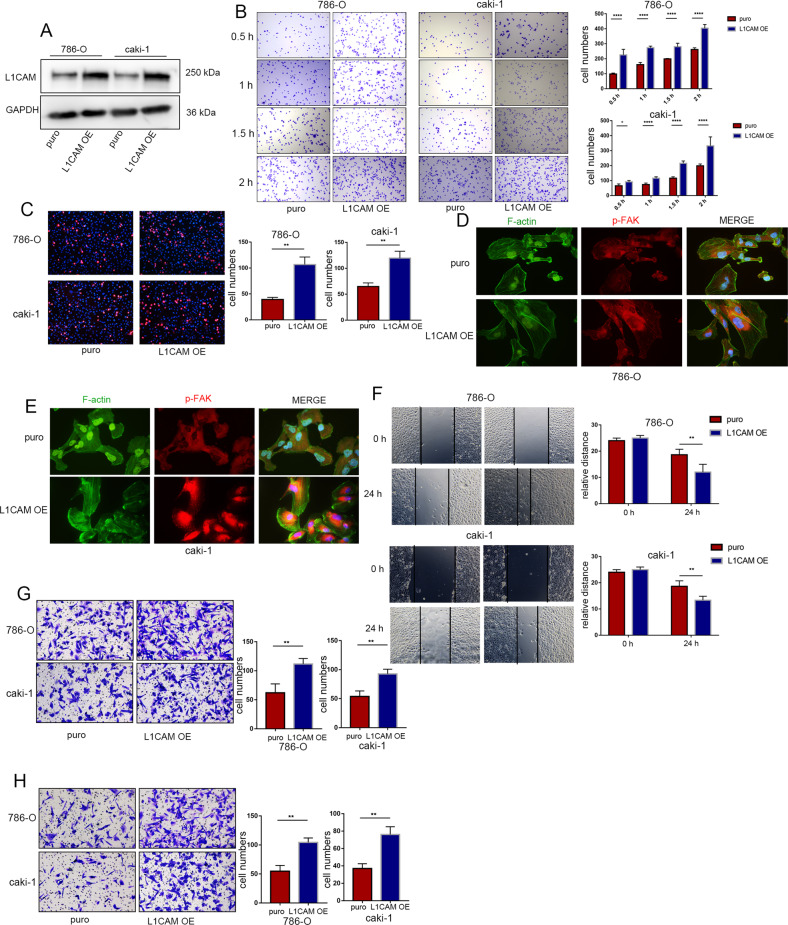
Overexpression of L1CAM promotes the adhesion, migration, and invasion abilities of RCC cells. **A** After infection with lentivirus, the L1CAM overexpression efficacy was confirmed in 786-O and Caki-1 cells using western blotting. **B** ECM adhesion assay was used to assess the effects of L1CAM overexpression on the ECM adhesion ability of 786-O and Caki-1 cells. The adherent cells were quantified in five randomly chosen fields. **C** A tumor-endothelial adhesion assay was used to assess the effects of 786-O and Caki-1 L1CAM overexpressing cells to evaluate cell-endothelial adhesion ability. The adherent cells were quantified in five randomly chosen fields. **D**, **E** Immunofluorescence of F-actin stress fiber formation and focal adhesion complex assembly in 786-O and Caki-1 cells after L1CAM overexpression (400× magnification). **F** Wound-healing assay showing the effect of L1CAM overexpression on the scratch repair activity of 786-O and Caki-1 cells. **G**, **H** Transwell migration and invasion analyses using 786-O and Caki-1 L1CAM overexpressing cells. The migrated and invaded cells were quantified in three randomly chosen fields. All data were presented as the mean ± SD. **P < 0.01, ***P < 0.001.

L1CAM-overexpressing lentivirus was used to establish an L1CAM overexpressing RCC cells model (Fig. 3A). Overexpression of L1CAM promoted the adhesion of RCC cells to ECM and vascular endothelial cells (Fig. 3B, C). L1CAM overexpression led to an increase in stress fibers and a compact cytoskeleton, with a parallel distribution of the majority of stress fibers throughout the cell observed (Fig. 3D, E). The migratory and invasive potential of RCC cells was significantly enhanced by L1CAM overexpression (Fig. 3F–H).

### The soluble ectodomain of L1CAM (L1CAM-ECD) drives RCC cell migration toward endothelial cells

**Fig. 4:**
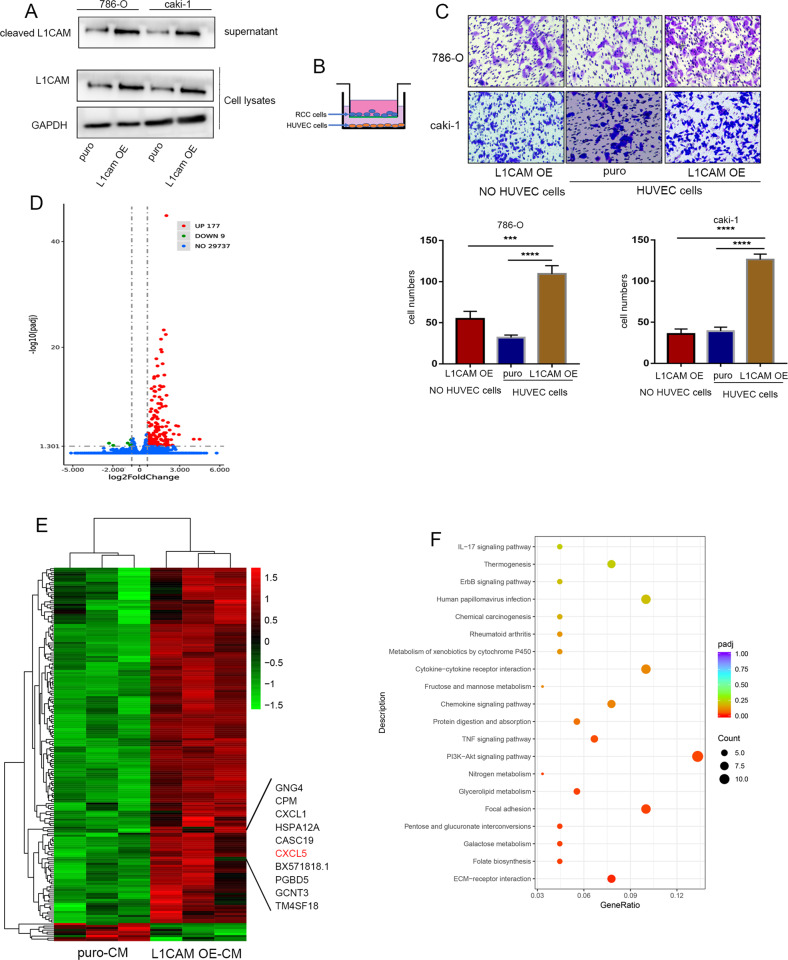
The soluble ectodomain of L1CAM drives RCC cell migration toward endothelial cells. **A** The soluble L1CAM-ECD levels in the culture supernatant were detected by western blotting after ultrafiltration using a 30-kDa ultrafiltration tube. **B**, **C** Transwell co-culture system of RCC and HUVEC and chemotaxis assays. RCC cells were seeded into 24-well Transwell upper chambers, and HUVEC were seeded into 24-well plates. After 6 h of incubation at 37 °C, the non-migrating cells were removed, and the number of migrated cells was randomly selected under the microscope from five fields of view. **D**, **E** Supernatants from L1CAM-overexpressing Caki-1 cells and control cells were collected after 48 h of culture. HUVECs were cultured in conditioned media for 24 h, followed by transcriptome analysis. Differentially expressed genes (DEGs) were identified and visualized using volcano plots and heat maps. **F** KEGG pathway analysis of the DEGs.

L1CAM was proteolyzed by ADAM10 and ADAM17 to release it from the cell membrane to form soluble L1CAM-ECD [23]. We speculated that L1CAM-ECD might mediate paracrine crosstalk between RCC and vascular endothelial cells. The expression of L1CAM-ECD in the culture supernatant was determined after ultrafiltration using a 30-kDa ultrafiltration tube. We observed that L1CAM overexpression in RCC cells led to increased L1CAM-ECD levels in the culture supernatant (Fig. 4A). A Transwell assay was used to investigate the role of L1CAM-ECD. Briefly, vascular endothelial cells and HUVECs were placed in the lower chamber, and RCC tumor cells were placed in the upper chamber of the Transwell chamber (Fig. 4B). This showed that endothelial cells induced elevated transpose migration in LICAM overexpressed RCC cells (Fig. 4C). Next, we collected supernatants from L1CAM-overexpressed cultures or control Caki-1 cells as conditioned medium at 48 h. HUVECs were incubated for 24 h in conditioned media and subjected to RNA-sequencing (RNA-seq) transcriptome analyses. We observed that 177 genes were upregulated, and nine were downregulated in HUVECs (Fig. 4D, E). KEGG pathway analysis revealed that “Cytokine−cytokine receptor interaction,” “Chemokine signaling pathway,” “Focal adhesion,” and “ECM-receptor interaction” were significantly elevated (Fig. 4F). Vascular endothelial cells release abundant cytokines and chemokines in response to L1CAM-ECD.

### CXCL5/CXCR2 axis promotes the directional migration of RCC cells

**Fig. 5:**
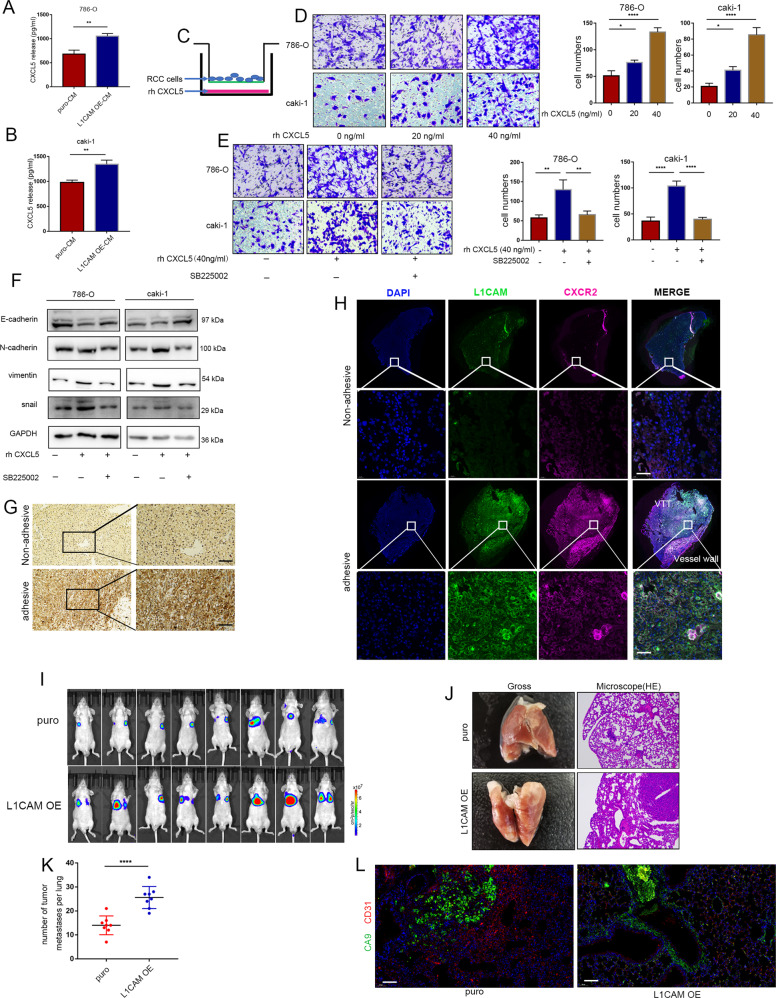
The CXCL5/CXCR2 axis promotes the directional migration of RCC cells. **A**, **B** The levels of CXCL5 secreted by HUVEC were detected using a human CXCL5 ELISA kit after 24 h of culture in a conditioned medium. **C**, **D** To determine the chemotactic effect of CXCL5 on RCC cell migration, 786-O and Caki-1 cells were resuspended in the upper compartment. CXCL5 was diluted to 0, 20, or 40 ng/mL and placed in the lower compartment, followed by incubation for 6 h at 37 °C, and the chemotaxis of CXCL5 to RCCs was analyzed. **E** To determine the effect of CXCL5-CXCR2 chemotaxis, RCC cells were pre-incubated with the CXCR2 inhibitor SB225002 (10 nM) for one hour and evaluated for chemotaxis. The number of migrated cells was quantified in three randomly chosen fields. **F** Changes in the expression of EMT proteins in RCC cells after treatment with 40 ng/mL of exogenous CXCL5 and SB225002. **G** Representative images of IHC staining of CXCR2 in noninvasive and invasive VTT tissue adjacent to the vessel wall sample (40× and 200× magnifications). **H** Panoramic scanning was performed for L1CAM and CXCR2 in paired VTT tissues after immunofluorescence staining. **I** Luciferase-labeled L1CAM overexpressing and control 786-O cells were injected into the tail veins of nude mice. The lung metastasis was monitored using an in vivo imaging system. **J** Gross lungs of nude mice and representative images of HE staining of metastatic nodules in the lungs of nude mice (100× magnification). **K** Number of lung metastases in each group. **L** Immunofluorescence staining for CA9 and CD31 in the small metastatic foci in the lungs. Data were presented as mean ± SD (400× magnification). Scale bars represent 50 μm. *P < 0.05, **P < 0.01, and ****P < 0.0001.

Transcriptome sequencing demonstrated that CXCL5 was significantly upregulated in HUVECs after incubation with L1CAM-overexpressed conditioned media (Fig. 4E). ELISA confirmed an increase in the CXCL5 protein levels in the supernatants of HUVECs culture media after incubation with conditioned media (Fig. 5A, B). Next, we evaluated the chemotactic effects of recombinant human CXCL5 on RCC cells (Fig. 5C), suggesting a significant increase in RCCs cell migration after treatment with 40 ng/mL of exogenous CXCL5 (Fig. 5D). CXCL5 is one of the binding ligands of CXCR2 [25], which enhances the migration of RCC cells and promotes epithelial-to-mesenchymal transition (EMT), which is blocked by the CXCR2 inhibitor SB225002 (Fig. 5E, F). We also observed that the VTT tissue with vessel wall invasion showed strong positive staining for L1CAM (Fig. 5G), the L1CAM^high^/CXCR2^high^ RCCs subgroup was more prone to perivascular localization in the VTT, promoted VTT adherence to the vascular wall, and deployed the perivascular tumor niche (Fig. 5H). Because the perivascular tumor niche is highly associated with metastasis, luciferase-labeled L1CAM overexpressing and control 786-O cells were injected into the tail veins of nude mice (*n* = 8, each group). It was observed that the number of lung metastases in the L1CAM overexpressing group was significantly higher than that in the control group (Fig. 5I–K). To further demonstrate the role of L1CAM in promoting the tropism of RCCs to vascular endothelial cells, the vascular endothelial cells were labeled using CD31 immunostaining. CA9 is a biomarker of RCCs and has been used to label RCCs in small lung metastatic foci. Significant spreading of L1CAM-overexpressing cells along the trunk and branches of the vessels was observed. The control group exhibited adherent clonal growth and a highly pro-angiogenic phenotype (Fig. 5L), indicating that the L1CAM may mediated perivascular tumor niche promotes metastasis.

### L1CAM interacts with ITGA5 and elicits activation of signaling downstream of integrin α5β1

**Fig. 6:**
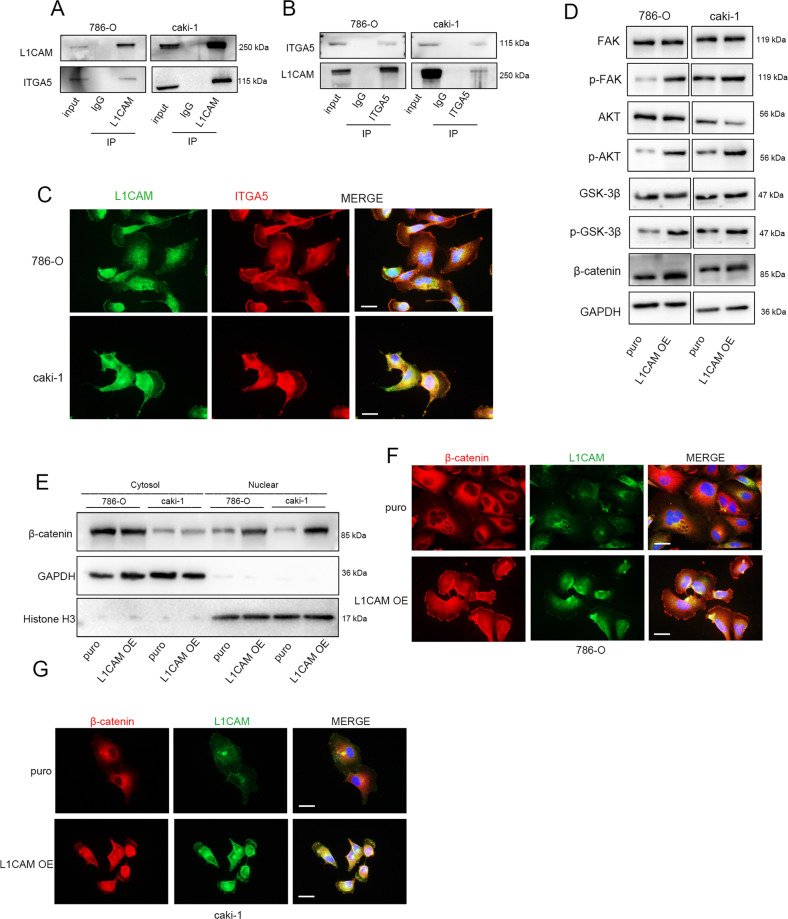
L1CAM interacts with ITGA5 and elicits activation of signaling downstream of integrin α5β1. **A**, **B** IP followed by western blotting. **C** Double staining with L1CAM and ITGA5 (confocal microscopy; 400× magnification). **D** Activation of protein kinases in integrin α5β1 signaling. **E** β-catenin expression in the cytoplasm and nucleus following L1CAM overexpression. **F**, **G** Immunofluorescence staining of β-catenin in L1CAM overexpressing RCC cells (400× magnification).

L1CAM has an RGD motif (-Arg-Gly-Asp-) in its sixth Ig domain [25], and the RGD domain is the most well-known functional domain of integrins. The BioGRID protein interaction database revealed that ITGA5 might be a binding ligand of L1CAM [26]. IP assays revealed that L1CAM interacted with ITGA5 (Fig. 6A, B), and immunofluorescence staining confirmed the colocalization of L1CAM and ITGA5 (Fig. 6C). ITGA5 belongs to the integrin α-chain family and binds to the β1 integrin subunit (ITGB1) to form the integrin α5β1 complex [25]. Integrin activation induces FAK phosphorylation, and PI3K/AKT is one of the downstream pathways of FAK integrin activation [25]. Glycogen synthase kinase-3-beta (GSK-3β) is an important AKT target gene that mediates β-catenin degradation and regulates the expression of β-catenin in the nucleus, thereby affecting the expression of downstream genes [25]. In 786-O and Caki-1 cells, phosphorylation of FAK, AKT, and GSK-3β was upregulated by L1CAM overexpression without affecting the total protein levels (Fig. 6D). We separated the cytoplasmic and nuclear proteins and observed that L1CAM overexpression increased β-catenin levels in the nucleus (Fig. 6D, E). Furthermore, the active role of L1CAM in the nuclear localization of β-catenin was confirmed by immunofluorescence (Fig. 6F, G).

### Inhibition of ITGA5 expression rescues the effects of L1CAM on the malignant behaviors of RCC cells

**Fig. 7:**
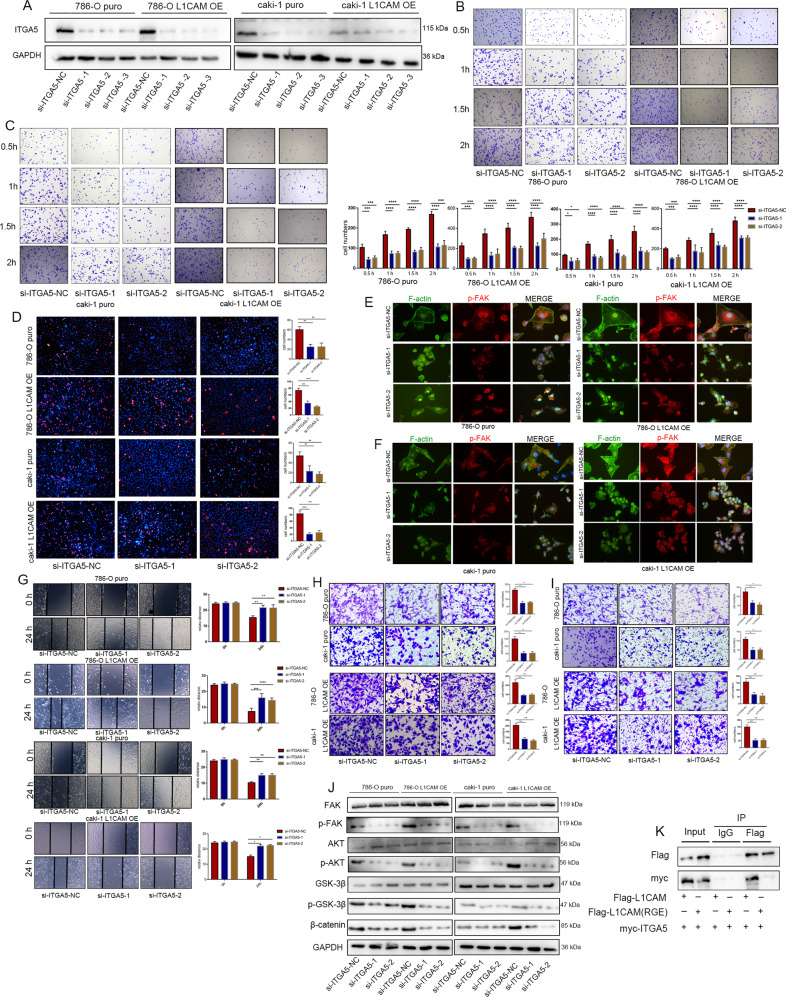
Inhibition of ITGA5 expression rescues the effects of L1CAM on the malignant behaviors of RCC cells. **A** Protein expression levels of ITGA5 in 786-O and Caki-1 cells after siRNA knockdown. **B**–**D** ECM adhesion and tumor-endothelial adhesion assays were used to assess the adhesion activity of RCC cells. **E**, **F** Immunofluorescence of F-actin stress fiber formation and focal adhesion complex assembly (400× magnification). **G**–**I** Wound healing, Transwell migration, and invasion assays were performed to assess the migration and invasion activity of RCCs. **J** Activation of protein kinases in integrin α5β1 signaling after inhibition of ITGA5 expression. **K** IP and Western blotting after co-transfection with FLAG-tagged L1CAM or FLAG-tagged L1CAM-RGE and Myc-tagged ITGA5. Data are presented as mean ± SD. *P < 0.05, **P < 0.01, ***P < 0.001, and ****P < 0.0001.

By using siRNA against ITGA5 to transfect L1CAM-overexpressing cells (Fig. 7A), We observed that inhibition of ITGA5 expression partially reversed the effect of L1CAM overexpression on promoting RCC cell adhesion to ECM and vascular endothelial cells (Fig. 7B–D). Interestingly, focal adhesion complex assembly induced by L1CAM overexpression was weakened by inhibition of ITGA5 expression (Fig. 7E). Next, we found that inhibition of ITGA5 reversed the enhanced migration and invasion abilities induced by L1CAM overexpression (Fig. 7G–I). Downregulation of ITGA5 also inhibited FAK, AKT, and GSK-3β phosphorylation caused by L1CAM overexpression. (Fig. 7J). These results suggest that ITGA5 is a downstream molecule of L1CAM and is involved in the progression of RCC.

### RGD motif is required for L1CAM to promote the adhesion, migration, and invasion abilities of RCC cells

RGD motif-mediated ITGA5 binding was required to further validate the function of L1CAM. We constructed an RGD motif mutant plasmid (L1CAM-RGE) by transfecting HEK293T cells with FLAG-tagged L1CAM-RGE and Myc-tagged ITGA5. Mutations in the L1CAM RGD motif inhibited its interaction with ITGA5 (Fig. 7K). However, cell adhesion, migration, and invasion were not affected by L1CAM-RGE plasmid transfection, indicating that L1CAM requires an RGD motif to promote adhesion, migration, and invasion of RCC cells (Supplementary Fig. S1A–G).

### β-catenin is a key protein affecting ADAM17 transcription

**Fig. 8:**
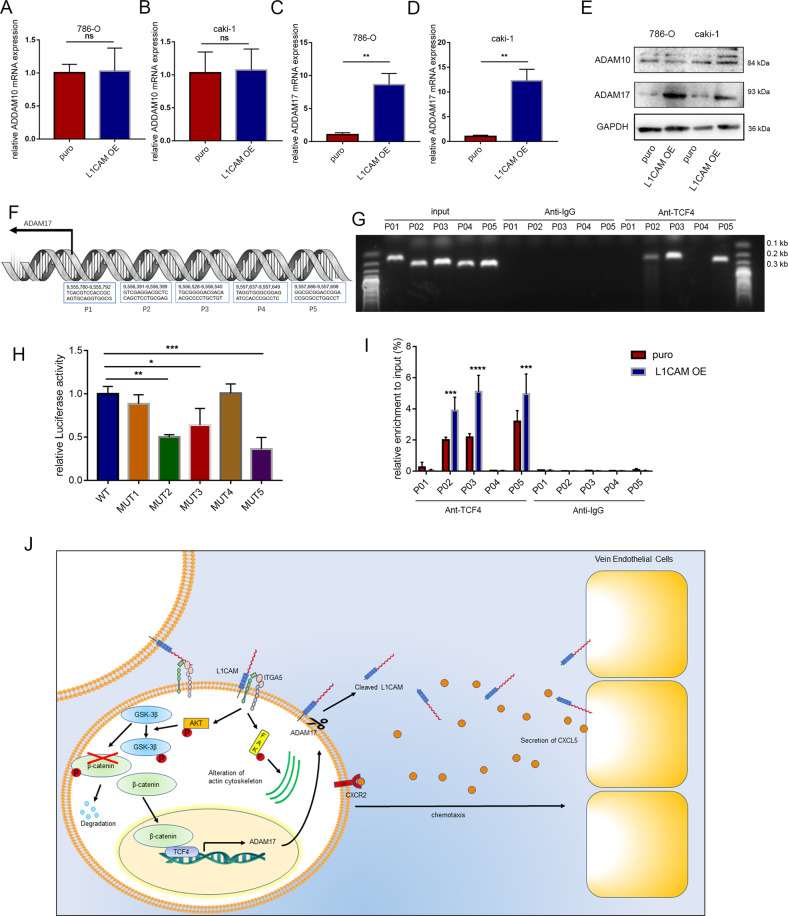
β-catenin is a key protein affecting ADAM17 transcription. **A**–**E** mRNA and protein levels of ADAM10 and ADAM17 after L1CAM overexpression. **F** Five TCF4 binding sites in the ADAM17 promoter. **G** ChIP assays were performed using TCF4 antibody in 786-O cells to detect the binding sites in the ADAM17 promoter regions. **H** Luciferase activity assay. **I** ChIP-qPCR assay. **J** L1CAM model deployed perivascular tumor niche promotes vessel wall invasion of tumor thrombus and metastasis of RCC. The data were presented as mean ± SD. ns no significance, *P < 0.05, **P < 0.01, and ***P < 0.001.

ADAM10 and ADAM17 play important roles in affecting L1CAM-ECD levels and the crosstalk between RCC and endothelial cells. The qPCR and western blotting showed that ADAM10 expression was not altered in RCC cells after L1CAM overexpression, whereas ADAM17 expression was significantly upregulated (Fig. 8A–E). L1CAM overexpression increased β-catenin levels in the nucleus, formed the β-catenin/TCF4 complex, and activated the transcription of downstream genes. The β-catenin/TCF4 complex may be a key factor that affects ADAM17 transcription. Several putative TCF4 binding sites between 2000 bp upstream and 200 bp downstream of the transcription start site of the human ADAM17 promoter were obtained from the JASPAR database, and five predicted high-scoring binding sites were selected for further study (Fig. 8F). We detected TCF4 binding sites 2, 3, and 5 are direct targets of TCF4 by ChIP and dual-luciferase reporter assays (Fig. 8G, H), and ChIP-qPCR showed that L1CAM promotes TCF4 enrichment on the ADAM17 promoter (Fig. 8I).

Overall, our study suggests that L1CAM promotes adhesion, migration, and invasion of RCC cells by interacting with ITGA5 and elicits activation of integrin α5β1 signaling downstream. L1CAM overexpression increases β-catenin levels in the nucleus to form the β-catenin/TCF4 complex. β-catenin/TCF4 complex activated transcription of ADAM17, which facilitates cleavage of the transmembrane ectodomain to release L1CAM-ECD. In response to L1CAM-ECD, endothelial-derived CXCL5 protein and its receptor CXCR2 promote the migration of RCC cells to endothelial cells and intravasation. The crosstalk between endothelial and tumor cells plays an active role in driving the metastatic spread of RCC (Fig. 8J).

## Discussion

Currently, radical nephrectomy combined with thrombectomy is the only effective treatment for RCC-VTT [27]. In the present study, we analyzed tissue samples of VTT adjacent to the vena cava wall from patients and demonstrated that L1CAM expression in VTT was significantly higher in the invasive part of the vessel wall compared to the noninvasive part. L1CAM mediates crosstalk between RCC and vascular endothelial cells and promotes VTT adhesion to the vascular wall and metastasis of RCC.

Recent studies have shown that L1CAM plays a crucial role in tumor progression. Specifically, the aggressive frontal regions of tumors express a large amount of L1CAM [28]. L1CAM is overexpressed in glioma stem cells and is an important factor in maintaining the growth and survival of glioma stem cells [29]. In vivo studies demonstrated that L1CAM promotes RCC metastasis to the lung, and RGD motif-mediated L1CAM and integrin α5β1 binding play a crucial role in this process.

ITGA5 belongs to the integrin α-chain family and binds ITGB1 to form the integrin α5β1 complex. ITGA5 plays a crucial role in cell surface adhesion and signaling. Integrin α5β1 binds to ligands and initiates cytoskeleton reorganization and the activation of downstream signals through intracellular signaling pathways, mediating cell adhesion, migration, and survival [30, 31]. These biological activities are mediated by RGD peptides, specific antibodies, and surface glycosylation [32]. ITGA5 expression is an important factor that affects the prognosis of non-small cell lung and breast cancers with bone metastases [33, 34]. ITGA5 mediates glioma cell dispersal and invasion through cell-matrix and cell–cell interactions [35]. In the present study, we observed that the binding of L1CAM to ITGA5 is critical for mediating the L1CAM function. L1CAM induces FAK signaling and assembly of focal adhesions by activating integrin α5β1, which increases the adhesion of RCCs. FAK phosphorylation activates PI3K/AKT signaling downstream, which ultimately inhibits β-catenin degradation and increases β-catenin expression in the nucleus, thereby enhancing the migration and invasion of RCC. Additionally, we observed that β-catenin interacts with TCF4 in the nucleus to initiate ADAM17 transcription, which causes LICAM cleavage at the membrane, thereby increasing the release and shedding of L1CAM- ECD.

Recent studies have indicated that endothelial cells may be a driving force for tumor metastasis. Endothelial-derived SLIT2 binds to the ROBO1 receptor in tumor cells and initiates cancer cell migration to endothelial cells and blood vessels. Inhibition of SLIT2 expression in endothelial cells has been shown to promote metastasis in breast and lung cancer mouse models [36]. We observed that vascular endothelial cells released several cytokines and chemokines in response to L1CAM- ECD. Among these, CXCL5 secreted by vascular endothelial cells plays a crucial role in mediating tumor cell migration to blood vessels. CXCL5 belongs to the CXC subfamily of chemokines and is involved in angiogenesis, tumor growth, and metastasis [37]. High CXCL5 levels in the serum are associated with tumor progression in nasopharyngeal carcinoma, lymph nodes, and distant metastasis [38]. The specific receptor for CXCL5 is CXCR2, which is overexpressed in cancers with high metastatic potential, such as breast, lung, and colon cancer cells [39–41]. In RCCs, CXCL5 binds to the CXCR2 receptor to promote EMT and enhance the migration of RCC cells. Our results demonstrated that L1CAM-ECD and CXCL5 mediate crosstalk between RCC and vascular endothelial cells. CXCL5 promotes RCC migration to the vascular endothelium. This crosstalk mediates the persistence of tumor cells in the perivascular niche; aids in oxygen, nutrients, and endothelial-derived paracrine factors; enhances tumor cell self-renewal, proliferation, and survival; and leads to metastasis formation.

Our study elucidates the important role of L1CAM in RCC progression. We observed a significant increase in L1CAM expression in VTT tissue in the invasive part of the vessel wall compared to that in the noninvasive part. High expression of L1CAM is closely associated with adhesion, migration, and invasion of RCC and augmented metastasis. Our results suggest that L1CAM deploys the perivascular tumor niche of RCC and is involved in the malignant process of RCC cells. Regulation of L1CAM expression may contribute to the prevention of RCC progression and treatment.

## Materials and methods

### RCC specimens and cell culture

The study protocol was approved by the Ethics Committee of Peking University Third Hospital. Informed consent was obtained from all patients prior to specimen and data collection. VTT samples with local invasion were collected, and samples without invasion or serious invasion were excluded. Twenty pairs of noninvasive and invasive VTT tissue samples adjacent to the vessel wall were collected from the same patients. RCC cell lines (786-O, Caki-1) and HEK293T cells were obtained from the American Type Culture Collection (ATCC), cultured in 1640 medium containing 10% fetal bovine serum (FBS, Biological Industries), whereas HUVECs cell lines were purchased from China Infrastructure of Cell Line Resource, and were grown in endothelial cell medium (ScienCell). The cell lines were identified by *STR* and tested for mycoplasma contamination.

### Establishment of stable cell lines, plasmid construction, and transfection

Human lentivirus-based short hairpin (sh)-L1CAM vector, empty lentiviral vector (sh-NC), and L1CAM overexpressing lentivirus were purchased from Genechem Co. Ltd. The L1CAM targeting shRNA sequences are listed in Supplementary Table S1. After infection of RCC cells with the lentivirus, cells were treated with 4 µg/mL puromycin and subsequently maintained in a medium containing 2 µg/mL puromycin. The si-ITGA5 was purchased from RiboBio Co. Ltd. (Guangzhou, China); corresponding sequences are shown in supplementary Table S2. We mutated leucine at position 556 (D) to lysine in the L1CAM mutant plasmids (L1CAM-RGE) to determine the role of the RGD motif in the L1CAM function. Luciferase reporter plasmids of the ADAM17 promoter region (−2000 to +200 bp) were constructed as pGL4.10-ADAM17-promoter plasmids. Further, plasmids or siRNAs were transfected using Lipofectamine 2000 (Invitrogen), according to the manufacturer’s protocol.

### Western blots and antibodies

Proteins were separated using 8–10% sodium dodecyl sulfate-polyacrylamide gel electrophoresis (SDS-PAGE), and Polyvinylidene fluoride (PVDF) membranes (Millipore, Billerica, MA, USA) were used for protein transfer. The PVDF membranes were incubated with primary antibodies overnight at 4 °C after blocking with a 5% blocking solution. After incubation with the appropriate secondary antibodies, the processing, exposure, and digital imaging was performed using Luminata Crescendo Western HRP substrate (Millipore). A full list of antibodies is provided in supplementary Table S3. The nuclear proteins were extracted using Cytoplasmic Protein Extraction Kit (Beyotime), according to the manufacturer’s instructions.

### Immunohistochemistry and immunofluorescence analysis

Antigens were recovered from the deparaffinized tissue sections and blocked with 5% bovine serum albumin. Tissue sections were incubated with primary and peroxidase-conjugated secondary antibodies overnight at 4 °C to detect antigen-antibody complexes. Thereafter, a color reaction was performed using a 3,3′-diaminobenzidine (DAB) substrate kit (ZsBio). Briefly, RCC cells were plated on coverslips and fixed with 4% PFA for immunofluorescence staining. The cells were treated with 0.25% Triton X-100 for 15 min and blocked with 5% donkey serum. The tissue sections were mixed with primary antibody overnight at 4 °C followed by multicolor immunofluorescence staining using the Treble-Fluorescence Immunohistochemistry Mouse/Rabbit Kit (Immunoway) according to the manufacturer’s protocol. Alexa 488 conjugated phalloidin (Beyotime) was used to stain F-actin.

### Cell adhesion assay

Collagen is the most abundant component of the ECM. RCC cells were plated on collagen I-coated 24-well plates at a density of 1 × 10^5^ per well and at pre-determined time intervals such as 0.5, 1, 1.5, or 2 h. The cells were washed to remove non-adherent cells, whereas adherent cells were fixed using 4% paraformaldehyde (PFA), followed by incubation for 10 min, and stained using crystal violet. Tumor-endothelial adhesion assay was performed as previously described [42], and HUVECs were seeded in 24-well plates and grown to confluence to investigate the adherence ability of RCC to vascular endothelial cells. The 1 × 10^5^ RCCs were labeled with the fluorescent dye DiL (Beyotime), seeded onto HUVEC monolayers, and allowed to adhere for 30 min. The cells were washed thrice with PBS to remove non-adherent RCCs, followed by DAPI counterstaining to visualize the nuclei. Images were captured using a fluorescence microscope.

### Wound-healing assay

To perform the wound-healing assay, RCCs were seeded in six-well plates and starved for 24 h. Further, a pipette tip was used to create a wounded cell monolayer. The wound closure process was observed for 24 h, and images were obtained.

### Transwell migration and invasion assays

RCCs (2 × 10^4^) and serum-free medium (100 µL) were added to the upper Transwell migration chamber (8-µm pore size; Corning), and 20% FBS medium was added to the lower chamber. After 24 h, cells were fixed with 4% PFA and stained using crystal violet. Cells were removed from the upper surface and counted under a microscope in five random fields. The upper chamber was coated with Matrigel (354480; Corning) before seeding the cells for the invasion assay.

### Immunoprecipitation

After centrifugation, the cells were lysed using IP lysis buffer, which was further incubated with antibodies (anti-L1CAM, ITGA5, or IgG as control antibodies) on a rotator overnight at 4 °C, followed by incubation with protein A + G agarose beads (Santa Cruz) for 4 h. Immune complexes were isolated from agarose beads and analyzed using SDS-PAGE, followed by immunoblotting. HEK293T cells were transfected with Myc-ITGA5, FLAG-L1CAM, or FLAG-L1CAM-RGE plasmids using Lipofectamine 2000 (Invitrogen) for 48 h followed by cell lysis. FLAG fusion proteins were immunoprecipitated using anti-FLAG magnetic beads (Beyotime), eluted with 3X FLAG peptide, and analyzed using SDS-PAGE, followed by immunoblotting.

### Co-culture and chemotaxis assays

RCC cells (2 × 10^5^ cells/mL in 200 µL of medium) were seeded into 24-well Transwell (8 µm pore PC membranes; Corning) upper chambers, and HUVEC were seeded into 24-well plates. After incubation for 6 h at 37 °C, the non-migrating cells were removed, and the bottom cells were fixed with 4% PFA and stained with crystal violet. The number of cells that migrated was randomly determined cells under the microscope from five different fields of view. To determine the chemotactic effect of CXCL5 on RCC cell migration, ~200 µL of 2 × 10^5^ RCCs were suspended in the upper compartment. CXCL5 (Novoprotein) was diluted to 0, 20, or 40 ng/mL and added to the lower compartment. After 6 h of incubation at 37 °C, the chemotaxis effect of CXCL5 on the RCCs was analyzed. RCC cells were pre-incubated with the CXCR2 inhibitor SB225002 (Selleck, 10 nM) for one hour to determine the effect of CXCL5-CXCR2 chemotaxis.

### Transcriptomic analysis

After 48 h of culture in a conditioned medium, the supernatants of L1CAM-overexpressing Caki-1 and control cells were collected and incubated with HUVECs for 24 h in conditioned media, followed by RNA-sequencing (RNA-seq) transcriptome analysis (Beijing Novogene Corporation). RNA-Seq libraries were generated using the NEB Next Ultra Directional RNA Library Prep Kit from Illumina (San Diego, CA, USA) according to the manufacturer’s protocol. The effect of soluble L1CAM on vascular endothelial cells was investigated using Illumina HiSeqTM 2500 sequencing according to the manufacturer’s guidelines (Illumina, CA, USA). Briefly, raw data were normalized, and clean reads were obtained from the raw data by excluding adapters, poly-N sequences, and low-quality reads to obtain high-quality cleaning data. Statistical analysis was performed for false discovery rate (FDR) and fold change filtering in altered mRNA following soluble L1CAM stimulation. Transcripts with *P* value ≤0.05 and threshold ≥1.5-fold change were considered statistically significant.

### RNA isolation and real-time PCR analysis

Total RNA was isolated using TRIZOL, and cDNA was synthesized using the transcriptor first strand cDNA synthesis Kit (Tiangen) using the manufacturer’s protocol. Real-time PCR was performed using the SYBR Green Master Mix (Yeasen). Primer sequences are provided in Supplementary Table S4.

### ELISA assay

The CXCL5 levels secreted by HUVECs were detected using a human CXCL5 ELISA kit (Elabscience), according to the manufacturer’s protocol.

### In vivo animal studies

Male BALB/c nude mice, aged 4–6 weeks, were used to evaluate the effects of L1CAM on tumor metastasis (The nude mice were randomly grouped, eight in each group, and no blinding was done). Nude mice experiments were approved by the Ethics Committee of Peking University Third Hospital. Briefly, 5 × 10^5^ L1CAM overexpressing 786-O cells and the respective control cells were injected into the lateral tail veins of nude mice. Metastatic progression was monitored weekly and quantified using a noninvasive bioluminescence In Vivo Imaging System 10 min after intraperitoneal injection of 4.0 mg of luciferin in 50 μl of saline, as previously described [43]. After eight weeks, the mice were sacrificed. Lungs of nude mice with distant metastasis were formalin-fixed and paraffin-embedded and sectioned at 5 mm throughout the organs, and one section in every 20 sequential sections was selected for hematoxylin and eosin staining.

### Chromatin immunoprecipitation (CHIP) assay

The ChIP assay was performed using the PierceTm Magnetic ChIP Kit (Thermo Scientific). 786-O cells were fixed with 1% formaldehyde, lysed using MNase, sonicated, and immunoprecipitated using a TCF4 antibody (Abcam, ab217668). After washing and reverse crosslinking, the precipitated DNA was amplified using primers, quantified using quantitative polymerase chain reaction (qPCR), and identified via agarose gel electrophoresis. Primer sequences are provided in supplementary Table S5.

### Luciferase reporter assay

HEK293T cells were transfected with a luciferase reporter gene (firefly luciferase), pRL-TK (Renilla luciferase plasmid), and TCF4 overexpressing or empty vector (pcDNA3.1) plasmid. After 48 h of transfection, luciferase activity was measured using a luminometer (Thermo Fisher Scientific) and a dual-luciferase reporter assay (Promega). Data represent the relative firefly luciferase activity normalized to Renilla luciferase activity.

### Statistical analysis

All experiments were conducted in triplicate, and data were expressed as means±standard deviation (SD). Data analysis was performed using GraphPad Prism (version 8.0). Statistical analysis was performed using an unpaired two-tailed *t*-test, one-way analysis of variance (ANOVA), and two-way ANOVA tests followed by Tukey’s multiple-comparison test, wherever necessary. Statistical significance was considered at *P* < 0.05.

## Supplementary information

Supplementary Figure 1

Supplementary Figure legends

Supplementary Tables

Original Data File

## Data Availability

All data generated or analysed during this study are included in this article and its supplementary information files.
