# Supplementary figures and images for: L1CAM deployed perivascular tumor niche promotes vessel wall invasion of tumor thrombus and metastasis of renal cell carcinoma
Source: Cell Death Discov. 2023 Apr 4;9:112. doi: 10.1038/s41420-023-01410-4 (PMC10073121; doi:10.1038/s41420-023-01410-4)

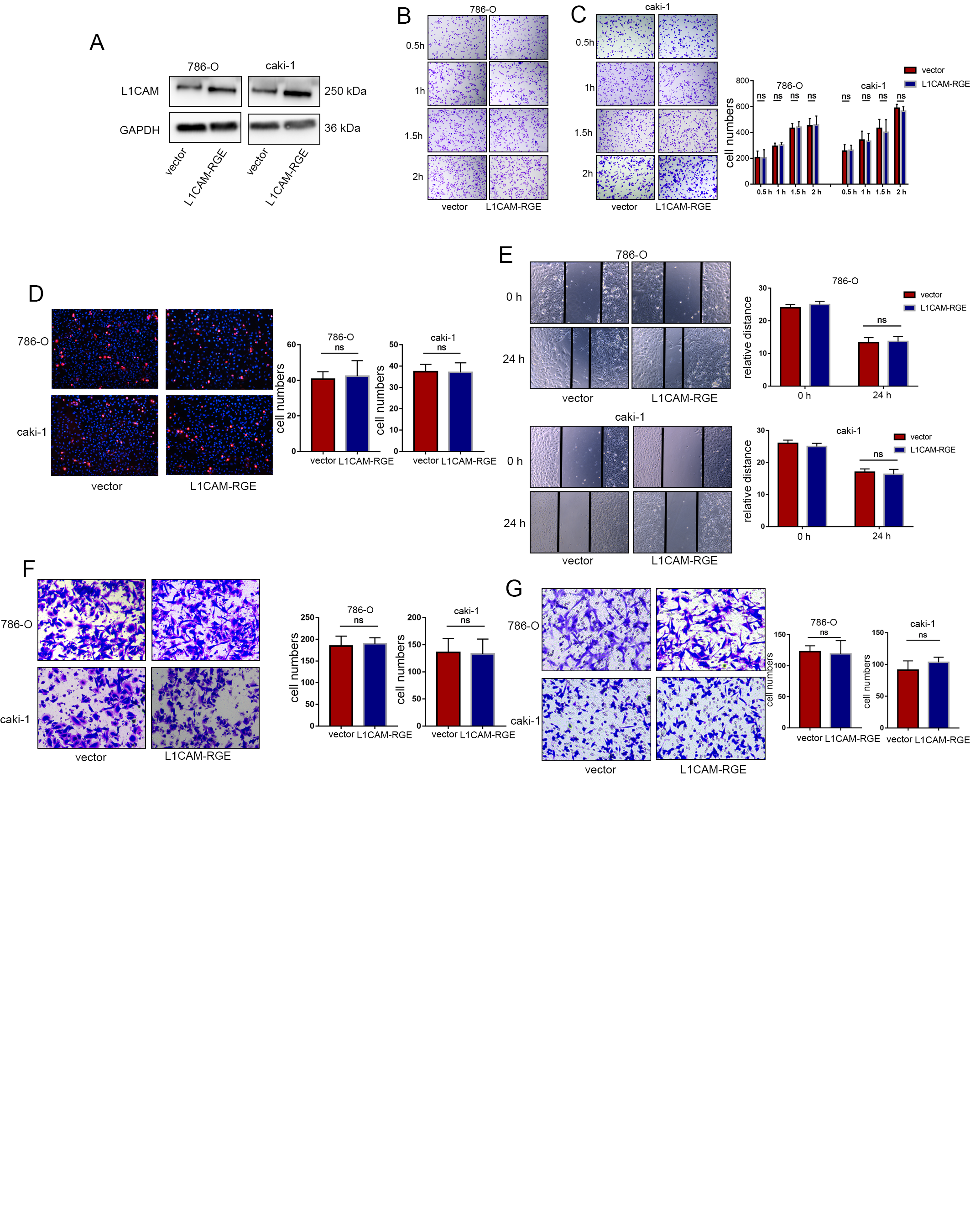

Supplement: Supplementary file 1 — Supplementary Figure 1 [file 41420_2023_1410_MOESM1_ESM.tif]

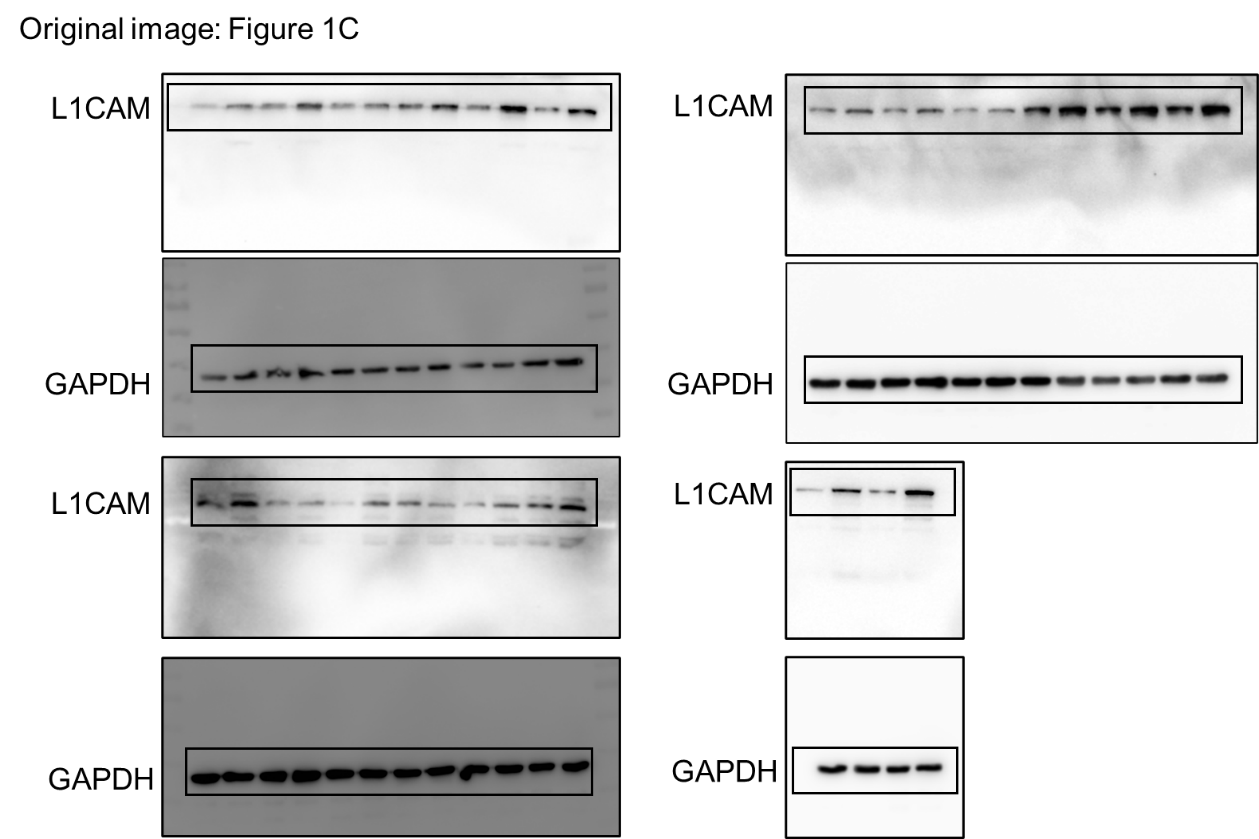


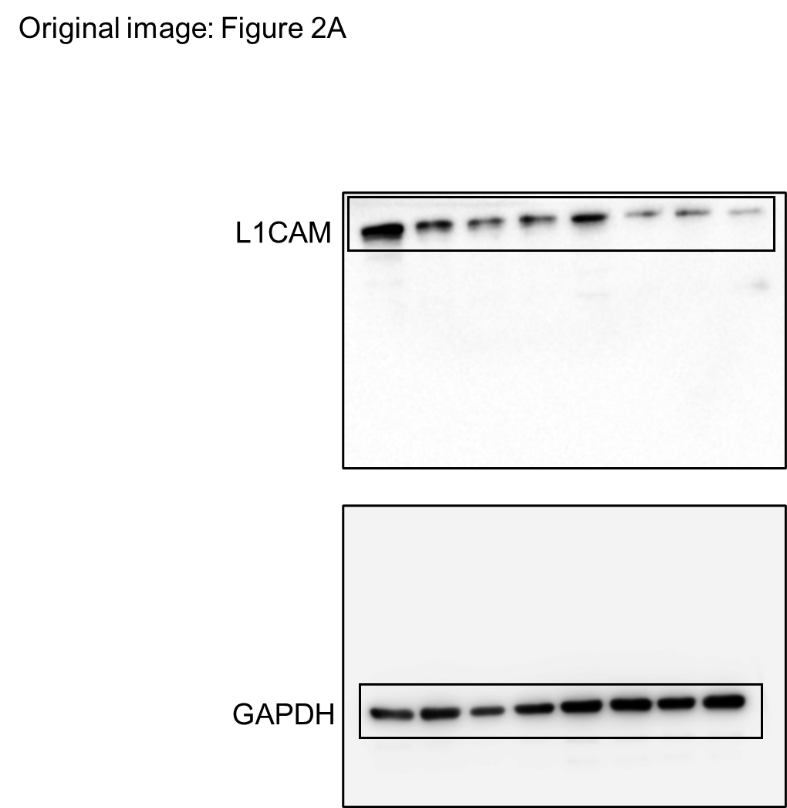

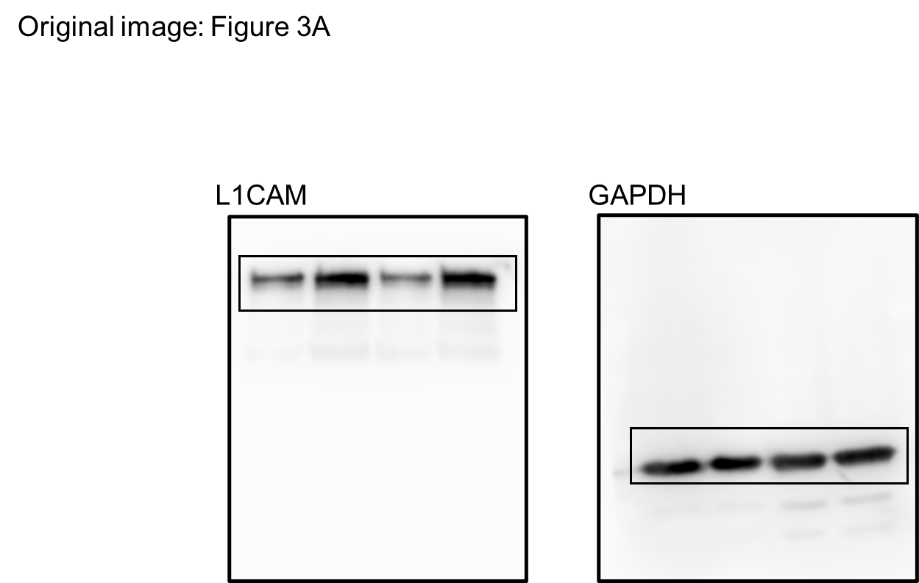


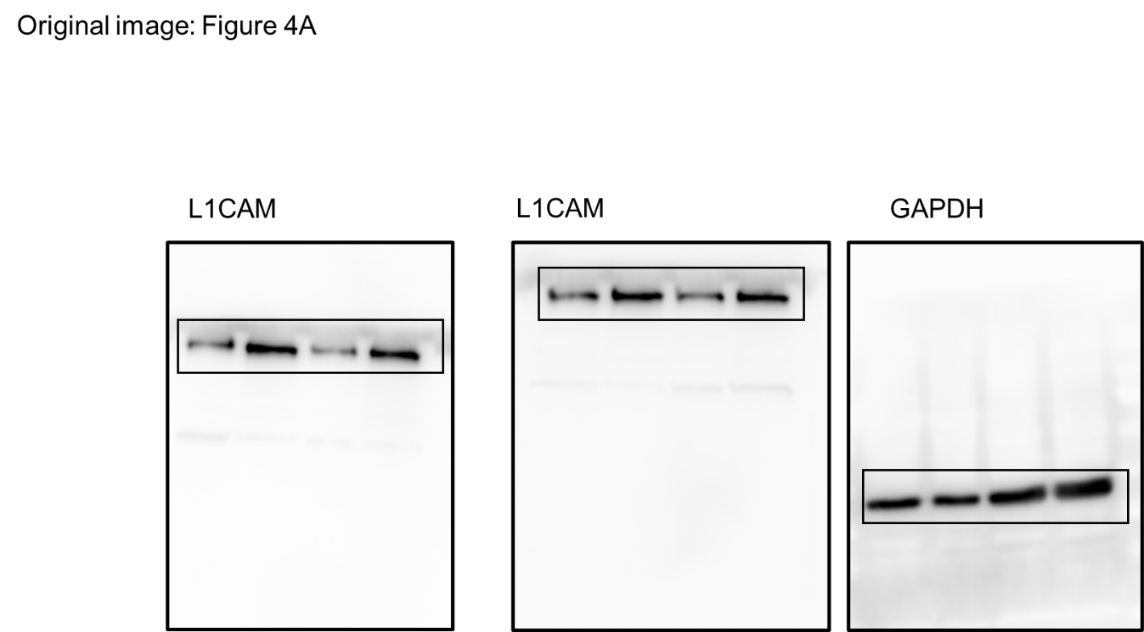


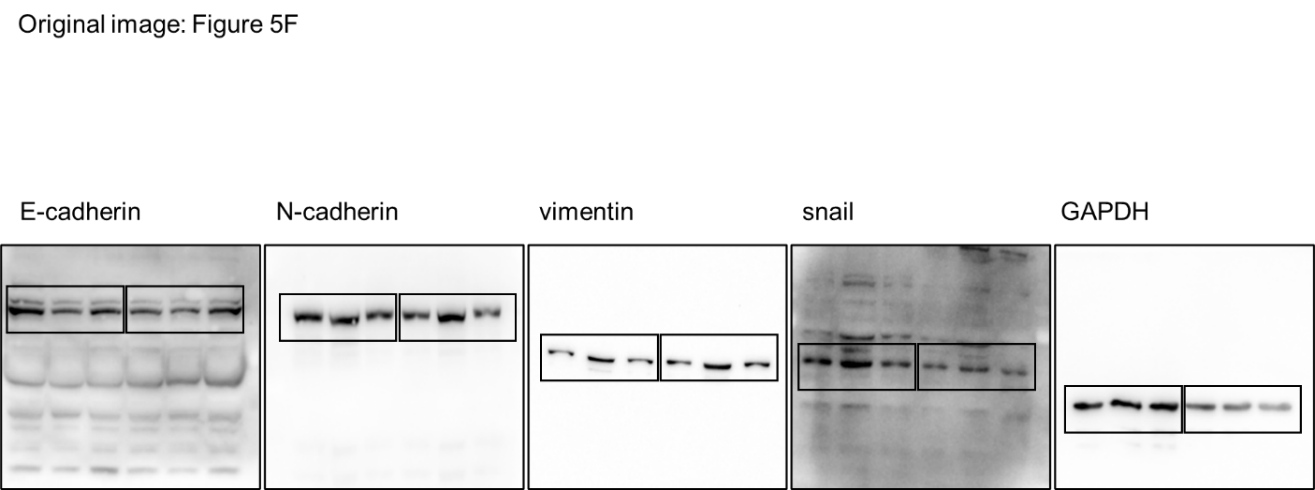


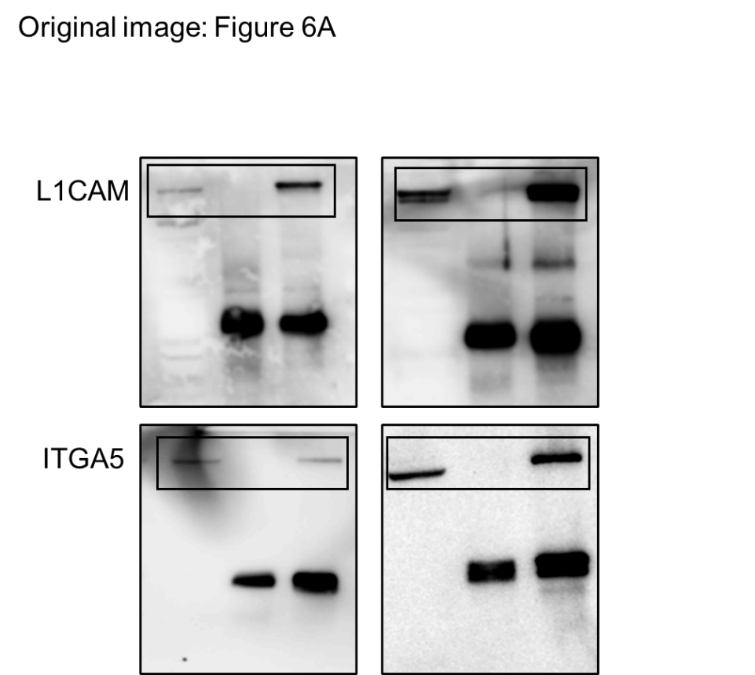


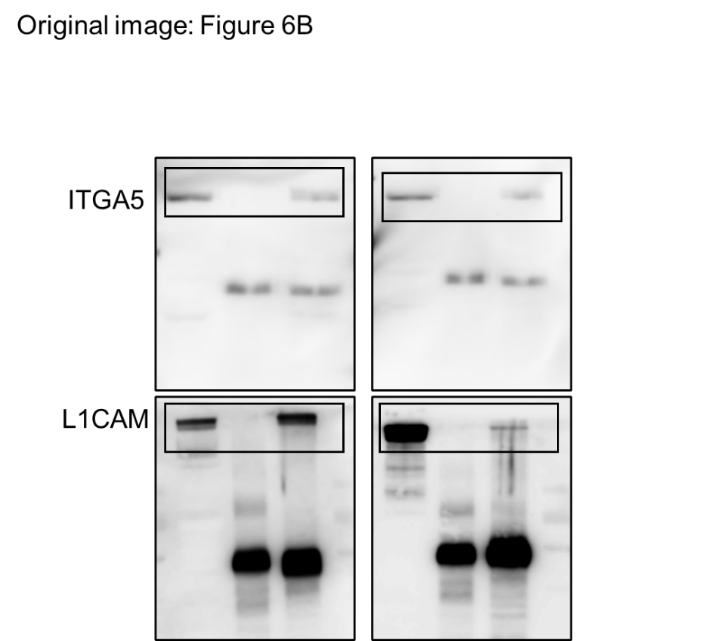


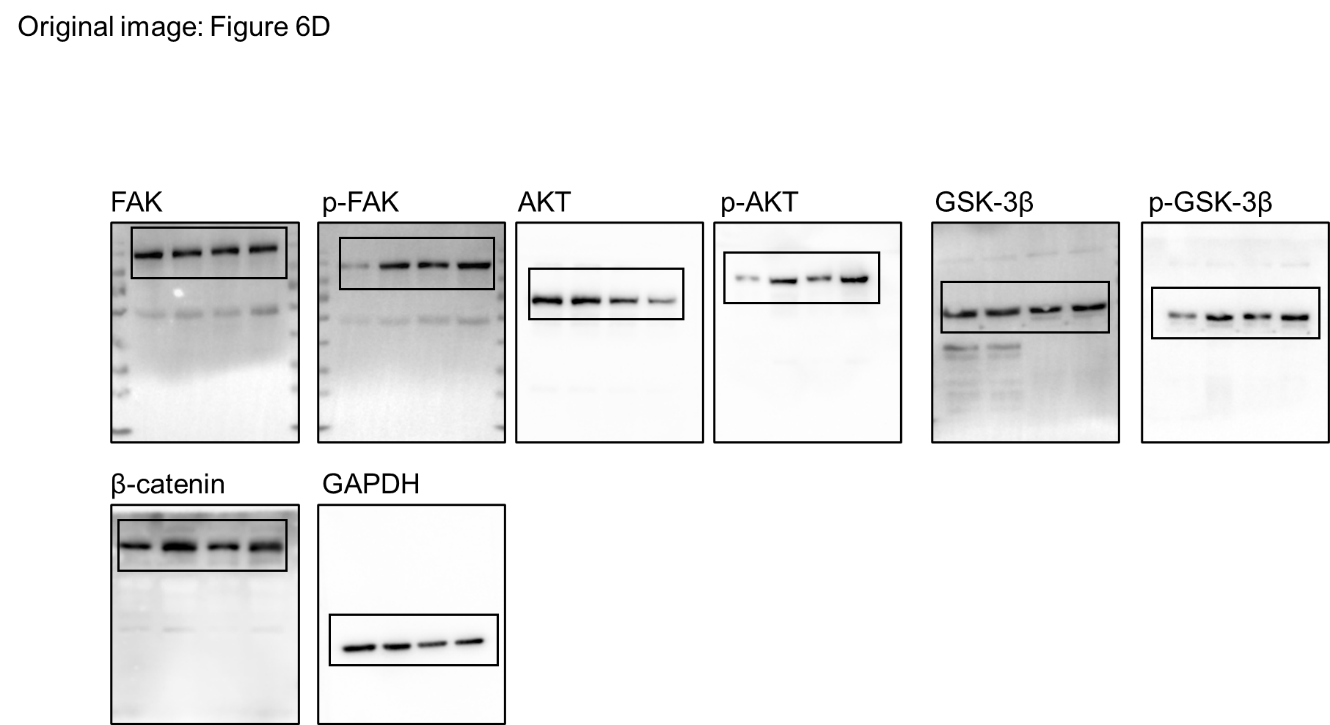


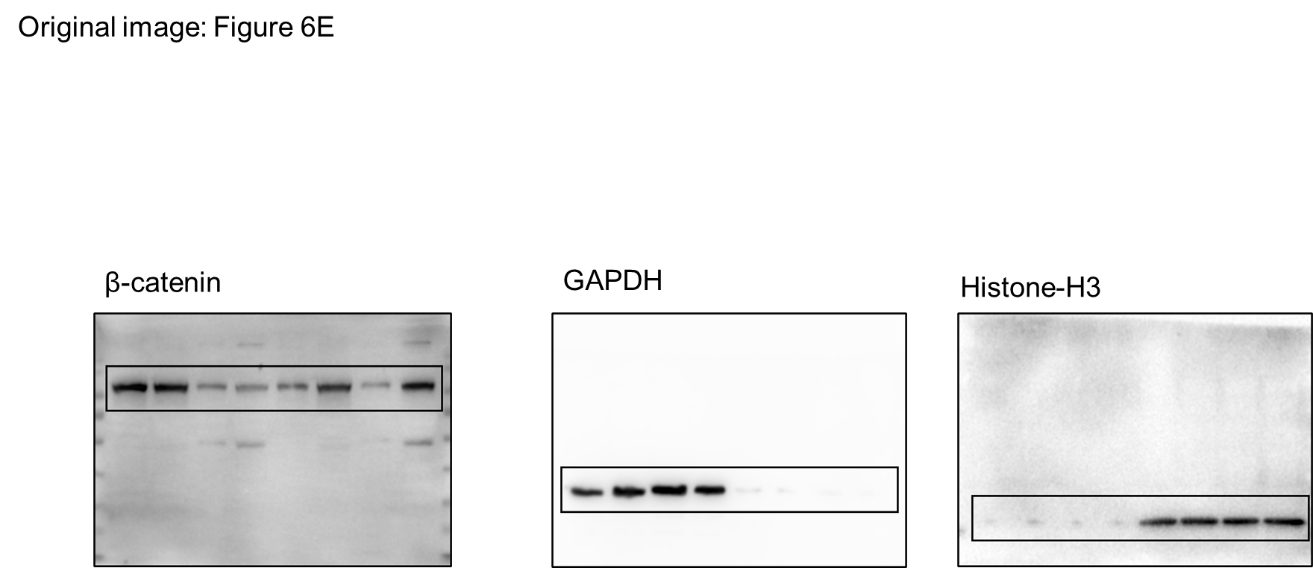


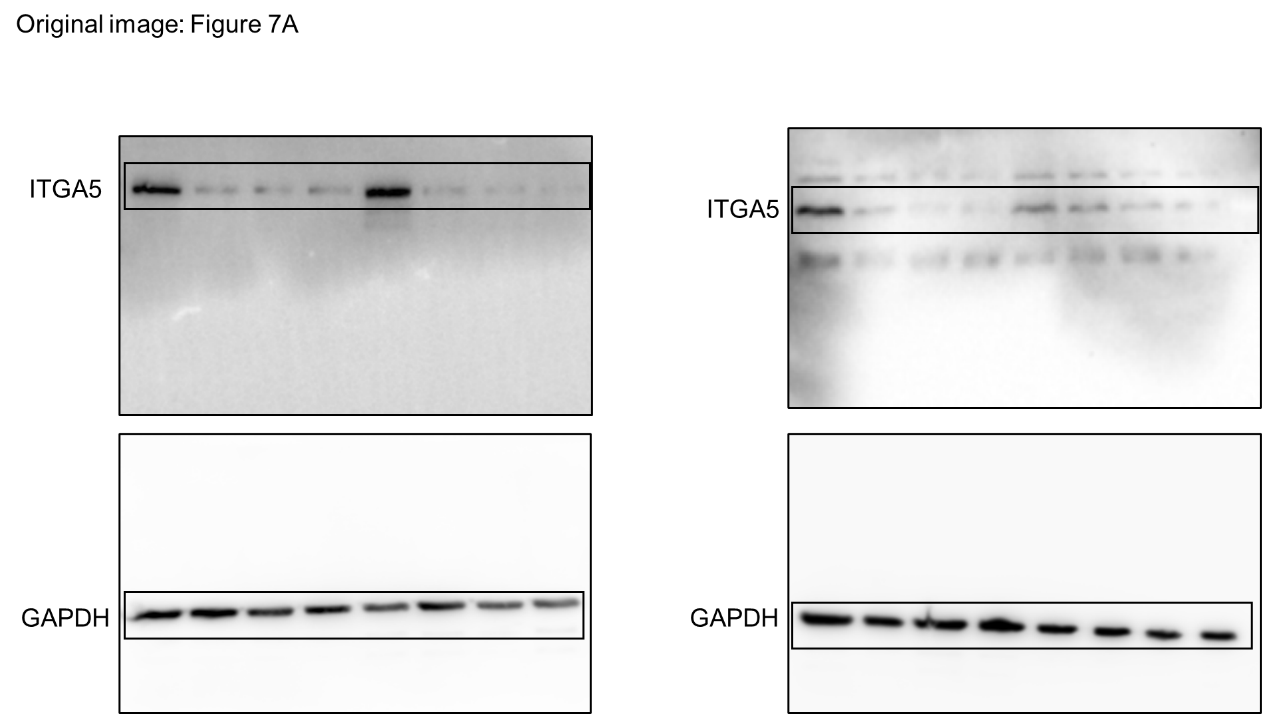


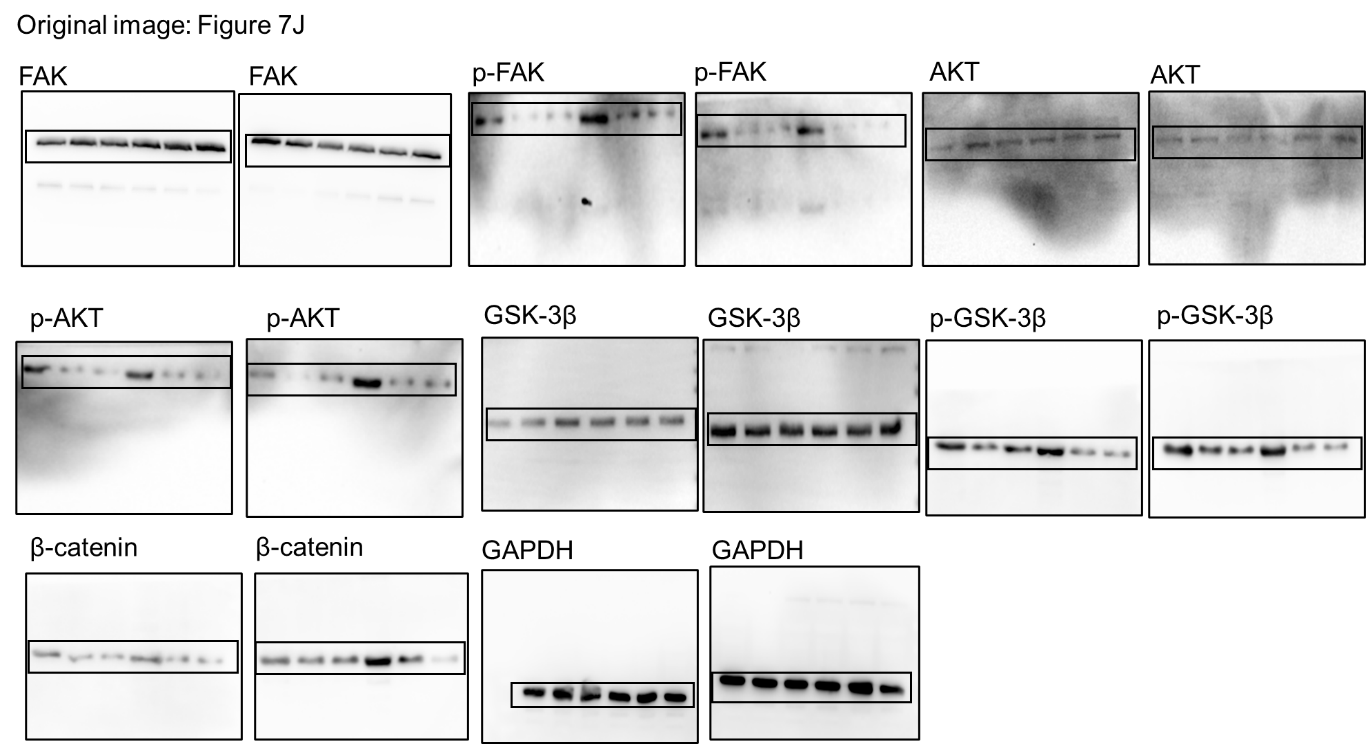


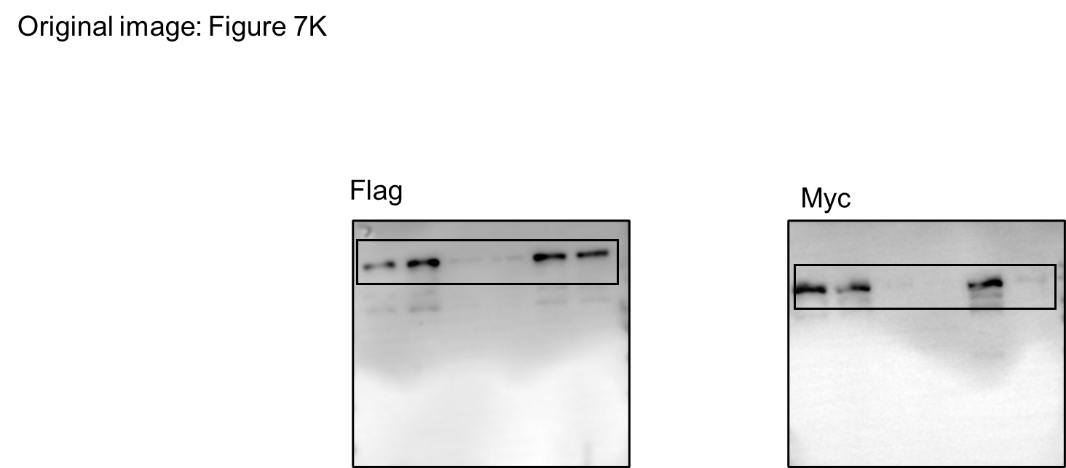


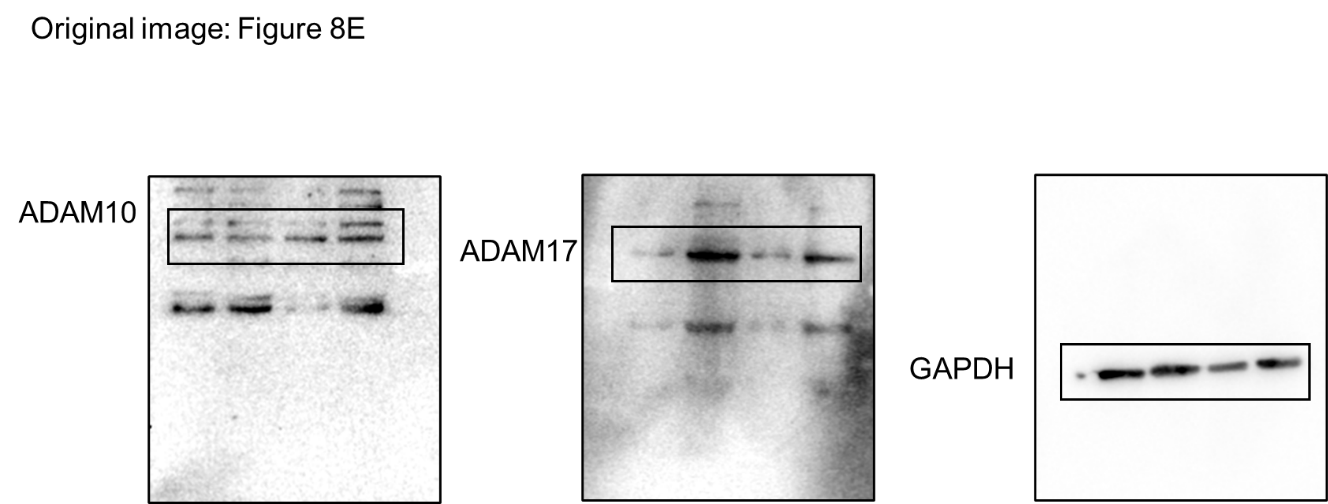


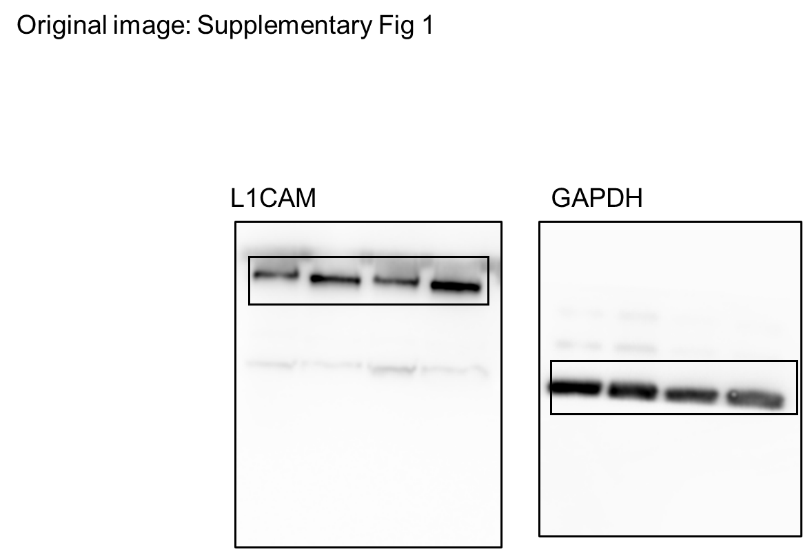

Supplement: Supplementary file 4 — Original Data File [file 41420_2023_1410_MOESM4_ESM.docx]
