# Supplementary material for: L1CAM deployed perivascular tumor niche promotes vessel wall invasion of tumor thrombus and metastasis of renal cell carcinoma
Source: Cell Death Discov. 2023 Apr 4;9:112. doi: 10.1038/s41420-023-01410-4 (PMC10073121; doi:10.1038/s41420-023-01410-4)
Supplement: Supplementary file 2 — Supplementary Figure legends [file 41420_2023_1410_MOESM2_ESM.docx]

**Supplementary Figure S1. RGD motif is required for L1CAM to promote the adhesion, migration, and invasion abilities of RCC cells**

(A) L1CAM expression after transfection with mutant L1CAM-RGE. (B-D) ECM adhesion and tumor-endothelial adhesion assays. (E-G) Wound healing, Transwell migration, and invasion assays. Data are presented as the mean ±SD. Ns: no significance.
