# Supplementary material for: L1CAM deployed perivascular tumor niche promotes vessel wall invasion of tumor thrombus and metastasis of renal cell carcinoma
Source: Cell Death Discov. 2023 Apr 4;9:112. doi: 10.1038/s41420-023-01410-4 (PMC10073121; doi:10.1038/s41420-023-01410-4)
Supplement: Supplementary file 3 — Supplementary Tables [file 41420_2023_1410_MOESM3_ESM.docx]

Supplemental Table S1.

| sh1 | CCGCTGCTTTGCCAGCAATAA |
| --- | --- |
| sh2 | CAGCAAGAGACATATCCACAA |
| sh3 | CGGCAACCTCTACTTTGCCAA |
| sh-NC | TTCTCCGAACGTGTCACGT |

Supplemental Table S2

| si-ITGA5-1 | sense: 5′- TCCTAGGTCTACTCATCTA-3', antisense: 5′-UCAGCUUGCUUCUUGUCGGTT-3'; |
| --- | --- |
| si-ITGA5-2 | sense: 5′- TGCTACCTCTCCACAGATA-3', antisense: 5′-UUCUCCAACUUUGCCACAGTT-3'; |
| si-ITGA5-3 | sense: 5′- CTCATCTCCGGGACACTAA-3', antisense: 5′-UGAGUGCGUUGUCCAGUUCTT-3'; |
| si-N.C | sense: 5′-UUCUCCGAACGUGUCACGUTT-3', antisense: 5′-ACGUGACACGUUCGGAGAATT-3'. |

Supplemental Table S3

| L1CAM | 1:1000, Abcam, ab270455 |
| --- | --- |
| ITGA5 | 1:1000, Proteintech, 27224-1-AP |
| FAK | 1:1000, Abcam, ab40794 |
| p-FAK | 1:1000, Abcam, ab81298 |
| AKT | 1:1000, Cell Signaling Technology, 9272 |
| p-AKT | 1:1000, Cell Signaling Technology, 4060 |
| GSK-3β | 1:1000, Cell Signaling Technology, 9315 |
| p-GSK-β | 1:1000, Cell Signaling Technology, 5558 |
| β-catenin | 1:1000, Cell Signaling Technology, 8480 |
| E-cadherin | 1:1000, Cell Signaling Technology, 96743 |
| N-cadherin | 1:1000, Cell Signaling Technology, 13116 |
| Vimentin | 1:1000, Cell Signaling Technology, 46173 |
| snail | 1:1000, Cell Signaling Technology, 3879 |
| ADAM10 | 1:1000, Abcam, ab124695 |
| ADAM17 | 1:1000, Abcam, ab39162 |
| GAPDH | 1:2000, Proteintech, 10494-1-AP |
| L1CAM | 1:200, Abcam, ab270455 (for IHC or IF analysis) |
| p-FAK | 1:200, Abcam, ab81298 (for IHC or IF analysis) |
| CXCR2 | 1:200, Proteintech, 20634-1-AP (for IHC or IF analysis) |
| CA9 | 1:200, Proteintech, 11071-1-AP (for IHC or IF analysis) |
| CD31 | 1:200, Immunoway, YT0752 (for IHC or IF analysis) |

Supplemental Table S4

| ADAM10 | Forward primer: 5'-ACCCTACAAATCCTTTCCGTTTCCC-3', Reverse primer: 5'- GCCAGACCAAGTACGCCATCATC-3' |
| --- | --- |
| ADAM17 | Forward primer: 5'- AGCAGATTCGCATTCTCAAGTCTCC-3', Reverse primer: 5'- GCAACATCTTCACATCCCAAGCATC-3'. |

Supplemental Table S5

| site 1 | forward primer: 5-ATCTTAAAAGTATGCACAATTGATAAATGAAAG-3, reverse primer: 5- GGCCTCCCAAAGTGCCAG-3; |
| --- | --- |
| site 2 | forward primer: 5-ACGCTTATAATCCTGGCACTTTGGG-3, reverse primer: 5- CCACTACAACCGACTGAACACCTC -3. |
| site 3 | forward primer: 5-GGTGGGAAGATTCCAGCTCTTGTAC-3, reverse primer: 5- GGCATTTGGTTCAGGGTCCTACTG -3; |
| site 4 | forward primer: 5-TCGTGACGACAGACGGATGGAG-3, reverse primer: 5-ACATGAATCGAGCCACAGAGTGC-3. |
| site 5 | forward primer: 5-TGGGTGGAGTTGGGACTCATACG-3, reverse primer: 5-CCAGAAGTGCAGGTGGCGTTAC-3; |
